# Stepwise Adipogenesis of Decellularized Cellular Extracellular Matrix Regulates Adipose Tissue-Derived Stem Cell Migration and Differentiation

**DOI:** 10.1155/2019/1845926

**Published:** 2019-11-06

**Authors:** Ziang Zhang, Rongmei Qu, Tingyu Fan, Jun Ouyang, Feng Lu, Jingxing Dai

**Affiliations:** ^1^Guangdong Provincial Key Laboratory of Medical Biomechanics, Department of Anatomy, School of Basic Medical Science, Southern Medical University, Guangzhou, China; ^2^Department of Plastic and Cosmetic Surgery, Nanfang Hospital, Southern Medical University, 1838 Guangzhou North Road, Guangzhou, Guangdong, China

## Abstract

Microenvironmental factors can modulate the cellular status of adipose tissue-derived stem cells (ASCs). In response to microenvironmental changes, cells can remodel extracellular matrix (ECM) proteins, which play an important role in regulating cell behaviors. During adipogenic differentiation, ECM components secreted from ASCs remodel dramatically. To evaluate the role of stepwise adipogenesis-induced cellular secretion of ECM on the behavior of ASCs, we cultured ASCs in growth and adipogenic media, and ECM secreted from cells was characterized and decellularized. The ASCs were then reseeded on decellularized ECM (d-ECM) to determine the regulatory effects of ECM on cellular behaviors. During adipogenesis, cell-secreted ECM underwent remodeling characterized by conversion from fibronectin-rich ECM to laminin-rich ECM. The cellular status of ASCs was tested after reseeding on decellularized ECM. When reseeded on growth d-ECM, ASCs exhibited greater migration ability. In contrast, ASCs seeded on adipogenic d-ECM underwent adipogenic differentiation. In addition, integrin subunit *α*v and integrins *α*6 and *α*7 were detected at significantly greater levels in ASCs cultured on growth and adipogenic d-ECM, respectively, suggesting that integrins play an important role in ASC migration and adipogenesis. This study demonstrated that stepwise adipogenesis-induced ECM production plays an important role in ASC migration and differentiation. In addition, this study provided a strategy to achieve precise regulation of stem cell function in adipose tissue engineering.

## 1. Introduction

Adipose tissue-derived stem cells (ASCs) originate from a wide range of sources and exhibit multilineage differentiation ability [1]. In plastic and reconstructive surgery, ASCs are beneficial for stem cell therapy and adipose tissue regeneration. In these processes, the use of a combination of biomaterials and adipose-derived stem cell transplantation results in better outcomes [2, 3]. However, the control of ASC differentiation into specific cell lineages is a significant challenge. Improved development of biomaterials for adipose tissue engineering requires elucidation of the mechanisms underlying regulation of ASCs to allow for precise and effective regulation of stem cell function.

To modify the surfaces of biomaterials to allow for the regulation of cellular behaviors, it is important to fully understand the environment in which cells are grown *in vivo*. In adipose tissue, ASCs are found within the stem cell niche, a specialized microenvironment that supports cellular homeostasis [4]. In the stem cell niche, various soluble factors and ECM proteins regulate cell behaviors [5]. Furthermore, ASCs are particularly regulated by cell ECM signaling. Previous studies have shown that self-renewal and differentiation of ASCs are tightly regulated by ECM organization and composition [6].

When the microenvironment is altered, ASCs remodel and optimize their microenvironment, particularly the ECM, which provides critical physical scaffolds and biochemical signals to maintain and regulate cellular functions of ASCs such as migration, proliferation, and differentiation [7]. During differentiation, the composition of the ECM changes dynamically [8, 9]. In addition, the phenotypes of ASCs are strongly influenced by the ECM composition from the proliferation stage through the differentiation stage [10]. Due to the importance of the ECM on the control of cellular behaviors, we hypothesized that ECM secreted from cells at different stages provides developmental stage-specific cues that direct cellular behaviors.

In this study, we examined ECM produced by ASCs during adipogenic differentiation. The ECM deposited by ASCs cultured on tissue culture plastic (TCP) was characterized after decellularization, and the effects of cell-derived ECM on ASC proliferation, migration, and differentiation were investigated. We also analyzed cell ECM interaction-related gene expression of integrins in reseeded ASCs.

## 2. Materials and Methods

### 2.1. Isolation and Cell Culture

Adipose tissue-derived stem cells (ASCs) were isolated from rat adipose tissue using a previously established method [11]. After isolation, ASCs were cultured on tissue culture plastic (TCP) dishes in Dulbecco's modified Eagle's medium/Ham's F12 (Gibco) supplemented with 10% fetal bovine serum (Gibco) and 1% penicillin/streptomycin (Gibco). Cells at passages 3-7 were used for experiments.

### 2.2. Adipogenic Differentiation and Oil Red O Staining

The ASCs were differentiated into the adipocytes by removing the growth media after 3 days and switching to adipogenic differentiation media (1 *μ*M dexamethasone, 10 *μ*M insulin, 0.5 *μ*M rosiglitazone, and 0.5 nM 3-isobutyl-1-methylxanthine) for a total of 14 days. After culturing for 14 days, the cells were washed three times with PBS, fixed with 5% paraformaldehyde for 5 min at room temperature, washed three times with PBS, and incubated for 5 min in distilled water. Then, the cells were incubated in Oil Red O solution (prepared by mixing three parts of 1 mg/ml stock solution in isopropanol with two parts of water, followed by filtration) for 10 min at room temperature. Stained cells were washed with PBS, and images were obtained using a phase contrast microscope (Primo Vert, Zeiss, Germany).

### 2.3. Decellularization and DNA Content Assay

To prepare cellular decellularized ECM from growth (growth d-ECM) and adipogenic media (adipogenic d-ECM), cellular components were removed by a previously reported method [12]. Briefly, ASCs were seeded at a density of 2 × 10^4^ cells/cm^2^ onto cell culture dishes (60 mm diameter) and cultivated for 7 days in the growth or adipogenic media, respectively. The media were changed every 2 or 3 days. For decellularization, the cells were washed with PBS and then removed by incubation with 0.5% Triton X-100 containing 20 mM ammonium hydroxide (NH_4_OH) in 0.1 M glycine in 1X phosphate-buffered saline (PBS) for 3 min at room temperature. After cell depletion, the samples were washed three times (5 min per time) with PBS and incubated for 5 min in distilled water. Next, the samples were treated with DNAse (100 *μ*g/ml) (Promega) and RNAse (100 *μ*g/ml) (Promega) at the same time for 1 h at 37°C. The samples were subsequently subjected to three freeze-thaw cycles (-80°C to 37°C) for 5 min per time. Then the samples were fixed in 0.1% glutaraldehyde for 6 h at 4°C and then treated with 0.1 M glycine in PBS. Then the cell-secreted extracellular matrix-coated TCP plates were stored at 4°C for later use.

Residual DNA after decellularization was tested using by DNeasy Kit™ (Qiagen, Valencia, CA) following previously established protocol [13]. Briefly, samples were prepared by using extract buffer DNeasy Kit™, then added in the working solution and incubated for 5-10 min at 37°C temperature in the dark. DNA content was then quantified with a microplate reader (Model 680; Bio-Rad) at 260 nm. The samples were normalized, and the concentration of DNA content was denoted as ng/ml.

### 2.4. BCA Assay

To quantitatively determine the total protein contents on growth and adipogenic ASCs secreted ECM before and after decellularization, samples prepared in 60 mm diameter plates were analyzed using BCA assay kit (Thermo Fisher Scientific). After rinsing three times with PBS, the samples were solubilized in RIPA cell lysis buffer (Sigma-Aldrich) with EDTA-free protease inhibitor cocktail (Thermo Fisher Scientific) and then collected in the 1.5 ml centrifuge tubes. Next, the samples were suspended and rocked gently for 30 min at 4°C and centrifuged at 12,000 g for 5 min. The supernatants were transferred into a new tube and subjected to BCA assay immediately. The total protein amount of each sample was normalized to the surface area of culture dishes, and thus, the concentration was denoted as mg/cm^2^.

### 2.5. Immunofluorescence (IF) Staining

ASCs were stained after 1 and 14 days of culture. The cells were washed three times with PBS and fixed with 4% formaldehyde for 10 min at room temperature. The cells were then permeabilized with 0.3% Triton X-100 for 2 min. The cells were blocked in 1% BSA for 30 min, then incubated with anti-fibronectin (ab2413; Abcam) and anti-laminin (ab11575; Abcam) primary antibodies overnight at 4°C. Then, the cells were incubated with secondary antibodies and counterstained with 4′,6-diamidino-2-phenylindole (DAPI). F-Actin was stained with phalloidin (Sigma-Aldrich) for 45 min.

### 2.6. Western Blot

The retention of fibronectin and laminin in non-/decellularization ASCs was measured by Western blot analysis as described previously [14]. Briefly, the sample lysates were prepared in RIPA buffer containing protease and phosphatase inhibitor cocktail (all from Thermo Fisher Scientific) as well as 1 mM phenylmethylsulfonyl fluoride (Calbiochem). Protein concentrations were measured using a BCA kit (Thermo Fisher Scientific), and equal amounts of protein samples were loaded onto reducing SDS-PAGE gels (Bio-Rad), separated by electrophoresis, and then transferred to PVDF membranes (Bio-Rad). After blocking with 5% skim milk, membranes were incubated overnight (4°C) with the following primary antibodies: anti-fibronectin (1 : 1000, Abcam), anti-laminin (1 : 1000; Abcam), and anti-GAPDH (1 : 2000 Abcam). Then the membranes were washed 3 times with PBST before following incubation with species-specific HRP-conjugated secondary antibodies. After another 3 washes with PBST, chemiluminescence detection was performed using an ECL kit (Thermo Fisher Scientific, USA). GAPDH served as internal controls to confirm that decellularized matrices did not contain cell-associated residuals.

### 2.7. Enzyme-Linked Immunosorbent Assay (ELISA)

Quantikine high sensitivity enzyme-linked immunosorbent assay (ELISA) kits (R&D Systems) were used to assess the expression of basic fibroblast growth factor (bFGF) and vascular endothelial growth factor (VEGF) in decellularized ECM according to the manufacturer's instructions. The absorbance of the wells at 450 nm was measured using a Multiskan MK3 microplate reader (Thermo Fisher Scientific, Waltham, Massachusetts). All samples were assayed in duplicate. A standard curve was constructed by plotting the mean absorbance of each standard against its concentration. The concentration of two growth factors was also normalized to the surface area of the culture dishes, and each growth factor amount was denoted as pg/cm^2^.

### 2.8. Cell Proliferation, Colony-Forming, and Migration Assays

To test cellular proliferation on different substrates, 2.5 × 10^3^ ASCs/cm^2^ ASCs were seeded on three 24-well culture dishes containing different substrates. After three days, cell proliferation was measured using the Cell-Light EdU Apollo 567 *In Vitro* Imaging Kit (RiboBio, Guangzhou, People's Republic of China) according to the manufacturer's instructions. The cells were counterstained with 4′,6-diamidino-2-phenylindole to stain the nuclei. 5-Ethynyl-2′-deoxyuridine incorporated into the 4′,6-diamidino-2-phenylindole in ASCs was detected. The ASC proliferation rate was assessed by counting the percentage of 5-ethynyl-2′-deoxyuridine-labeled ASCs in 4′,6-diamidino-2-phenylindole-labeled ASCs in 10 fields of each dish. Counting was performed by two blinded scientists.

Meanwhile, for colony-forming assay, undifferentiated ASCs were reseeded on TCP, growth d-ECM, and adipogenic d-ECM, respectively. The assay was modified from a previously described method [15]. Briefly, ASCs were transferred into differently coated 60 mm plates at the density of 1000 cells/well. After 5 h incubation at 37°C to allow cell attachment, the dishes were washed twice with PBS to remove nonadherent cells. One half of the medium was replaced every 3 days and was then subject to colony formation assay for 15 days. The colonies were stained with 0.5% crystal violet.

To test cellular migration on different substrates, 5 × 10^3^ ASCs/cm^2^ were seeded in culture dishes with three different substrates using Culture-Insert (Eubio 80241). Cell migration over time was monitored using phase contrast microscopy (Primo Vert, Zeiss, Germany) at 0, 12, and 24 h.

### 2.9. Gene Expression as Determined by Quantitative Real-Time Polymerase Chain Reaction (qRT-PCR)

RNA was isolated from reseeded ASC samples using Trizol Reagent (Invitrogen/Life Technologies, Carlsbad, CA) according to the manufacturer's instructions, followed by reverse transcription. RNA concentration was measured using a NanoDrop 2000 (Thermo Fisher Scientific, USA). Complementary DNA was synthesized using a RevertAid First Strand cDNA Synthesis Kit (cat. # K1621; Thermo Fisher Scientific, USA). qRT-PCR was performed using a StepOnePlus Real-Time PCR System (cat. # 4376600; Applied Biosystems/Life Technologies) using FastStart Universal SYBR Green Master (Rox) (cat. # 04913914001; Roche). Polymerase chain reaction specificity was assessed by the Ct method. Glyceraldehyde 3-phosphate dehydrogenase (GAPDH) was used as a housekeeping gene. Target genes and their primer sequences are summarized in Table 1.

## 3. Statistical Analysis

All data were analyzed using IBM SPSS version 20.0 software (IBM Corp., Armonk, NY, USA). Data are expressed as the means ± SD. Student's *t*-test was used to determine the levels of significance for comparisons between two independent samples. Comparisons among three or more groups were performed using one-way analysis of variance (ANOVA) with Tukey's post hoc test for direct comparisons between groups. Differences were considered statistically significant when ^∗^*p* < 0.05, ^∗∗^*p* < 0.01, or ^∗∗∗^*p* < 0.001.

## 4. Results

### 4.1. Characterization of ECM during Stepwise Adipogenesis of ASCs

**Figure 1:**
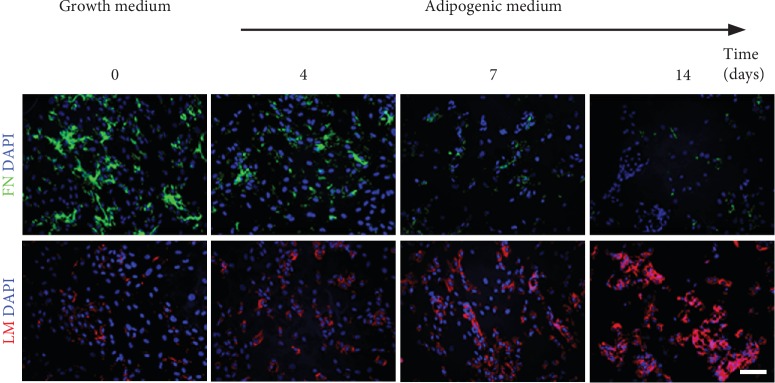
Immunostaining ECM proteins secreted during adipogenesis. FN: fibronectin; LM: laminin. Scale bar = 100 *μ*m.

Lipid droplets were visually observed in ASCs cultured in adipogenesis medium (AD) for 14 days, and no lipid droplets were observed in ASCs cultured in growth medium (GM) (Fig. [Supplementary-material supplementary-material-1]). Deposition of ECM proteins was detected by immunofluorescence analysis. Fibronectin was abundant in the ECM of ASCs cultured in growth medium. However, fibronectin decreased gradually in the ECM of ASCs undergoing adipogenesis and was only weakly detected in the ECM after 14 days. In contrast, laminin increased gradually during adipogenic differentiation but was only present at low levels in the ECM produced by undifferentiated ASCs (Figure 1).

### 4.2. Confirmation of Decellularization

**Figure 2:**
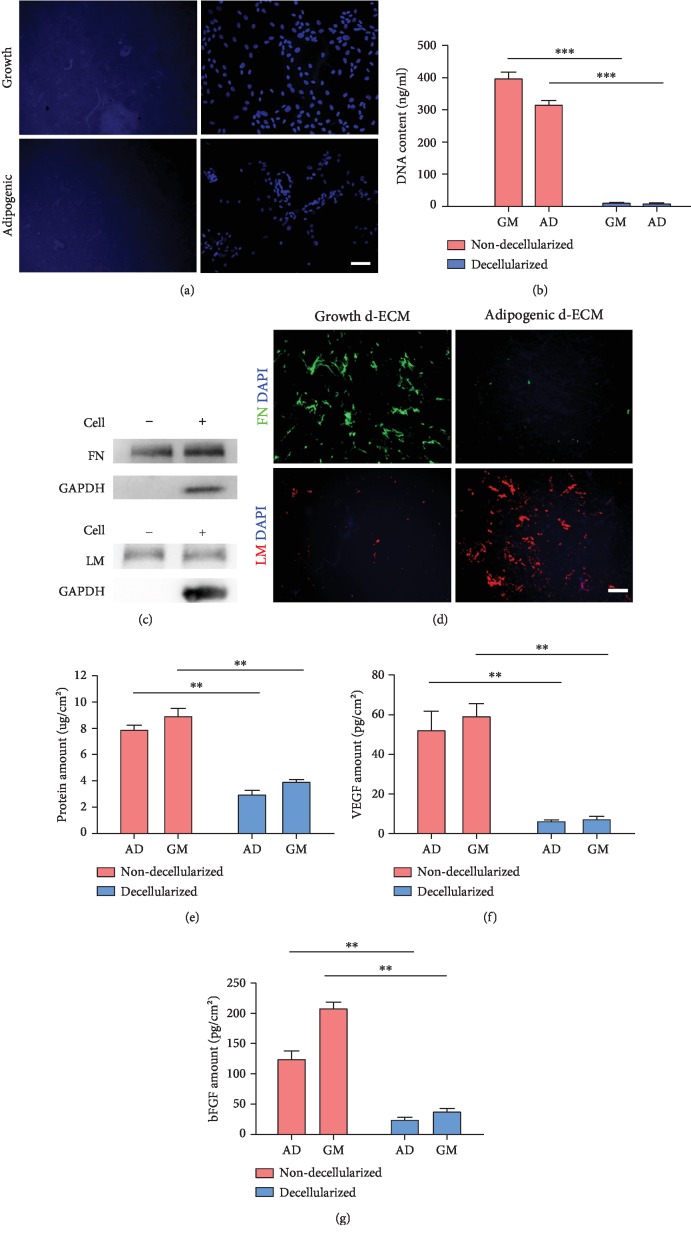
Confirmation of decellularization of ASC ECM. (a) DAPI staining. (b) DNA content was measured to confirm removal of nuclei. GM: growth media; AD: adipogenic media. (c) Western blot of GAPDH was performed to confirm removal of cell components. (d) Immunostaining analysis of ECM protein contents after decellularization. FN: fibronectin; LM: laminin. (e) Total protein content before and after decellularization was measured using the BCA assay. Total VEGF and bFGF contents were analyzed by ELISA. (f) VEGF. (g) bFGF. Results are presented as the mean ± SD; Student's *t*-test. ^∗∗^*p* < 0.01 and ^∗∗∗^*p* < 0.001, compared with two groups. Scale bar = 100 *μ*m.

ASCs were decellularized, and removal of cellular components was confirmed by nuclear staining with DAPI, which was visible in plates containing cells and was absent after decellularization (Figure 2(a)). The amount of DNA of the GM and AD groups was significantly decreased after decellularization (Figure 2(b)), and the intracellular protein GAPDH was absent, as determined by Western blot (Figure 2(c)); these results further confirmed that cellular material was all removed. After decellularization, ECM components such as fibronectin and laminin remained in the culture plates, also suggested by the fact that fibronectin was robustly detected in growth d-ECM but only weakly detected in adipogenic d-ECM, while the laminin showed a contrary trend (Figure 2(d)). Total protein was measured using the BCA assay, which showed that the total protein content was significantly decreased following decellularization (Figure 2(e)). The expression of two major cytokines, VEGF and bFGF, was significantly lower after decellularization (Figures 2(f) and 2(g)). These results indicated that decellularization removed cellular components and cytokines.

### 4.3. ASC Morphology and Proliferation on Decellularized ECM

**Figure 3:**
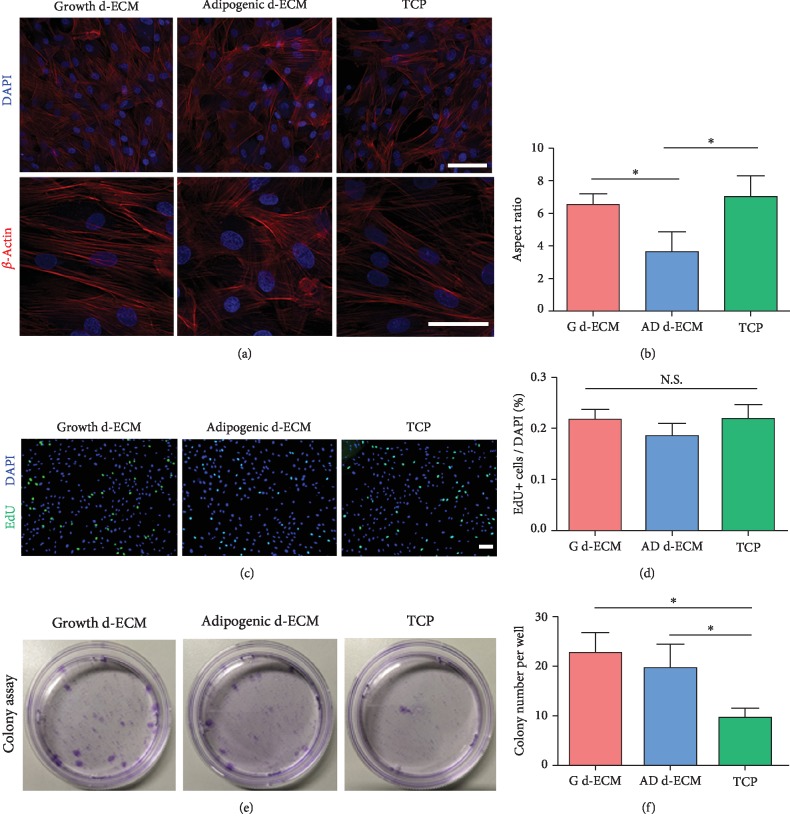
Cellular morphology and proliferation of ASCs cultured on different substrates. (a) Cellular morphology of ASCs cultured on different substrates. ASCs were reseeded on three different substrates: growth d-ECM, adipogenic d-ECM, and tissue culture polystyrene (TCP). Immunostaining for F-actin. (b) ASC morphology is quantitatively compared by measuring cell aspect ratio using ImageJ software. (c) EdU assay for proliferation of ASCs. (d) Quantitative analysis of EdU^+^ cell proliferation rate. (e) Colony-forming assay. (f) Quantitative analysis of colony number per well. Results are presented as the mean ± SD; one-way ANOVA followed by Bonferroni's post hoc test analysis for multiple comparison. Scale bar = 100 *μ*m.

To investigate the effects of d-ECM on cellular morphology, ASCs were reseeded on growth and adipogenic d-ECM. TCP without ECM was used as a control. After culturing for 3 days, the reseeded ASCs were stained for F-actin to evaluate cell morphology (Figure 3(a)). The ASCs reseeded on adipogenic d-ECM showed a less elongated, more rounded morphology than those reseeded on growth d-ECM. The ASCs grown on TCP had a longer spindle shape than those grown on d-ECM. To investigate the role of d-ECM on cell growth, proliferation of reseeded ASCs was analyzed using EdU staining, which showed that there were no significant differences in proliferation of ASCs grown on d-ECM or TCP (Figures 3(b) and 3(c)). However, colony-forming assay showed that ASCs reseeded on d-ECM exhibited better self-renewal properties than cells seeded on TCP (Figure 3(d)).

### 4.4. ASC Migration and Differentiation on Decellularized ECM

**Figure 4:**
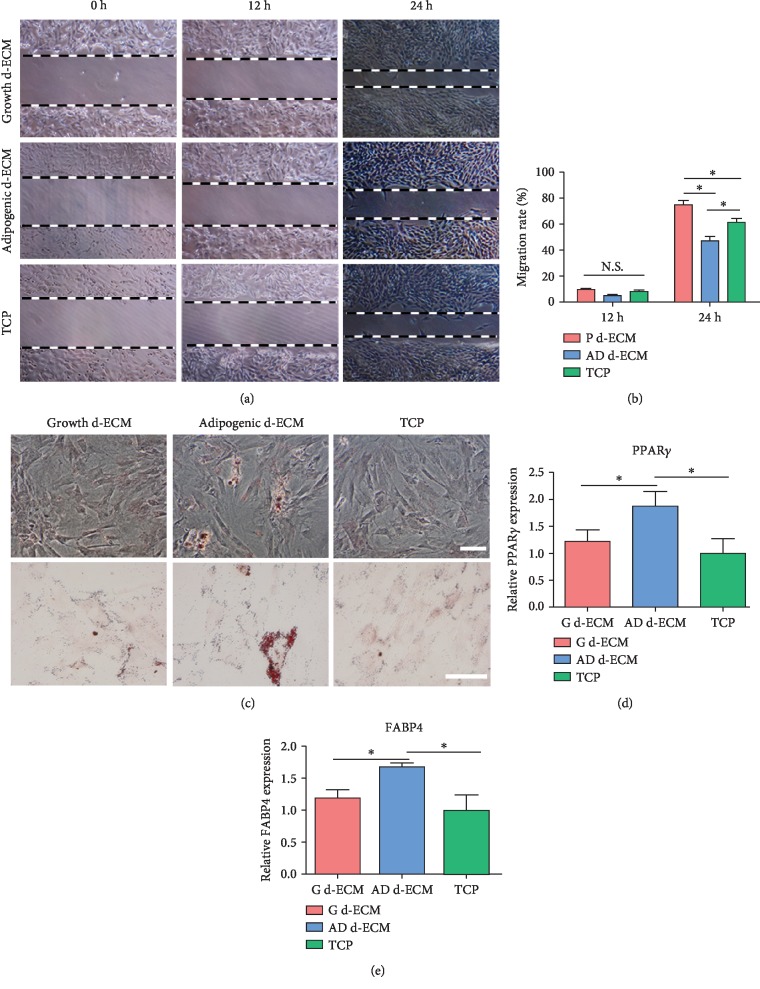
Migration and ability to undergo adipogenesis of ASCs on different substrates. (a) Migration ability of ASCs on three different substrates: growth d-ECM, adipogenic d-ECM, and tissue culture polystyrene (TCP). (b) Quantitative analysis of migration rate in all groups. (c) Oil red O staining for lipids in ASCs cultured on three different substrates. Gene expression of the adipogenic markers (d) PPAR*γ* and (e) FABP4. Results are presented as the mean ± SD. ^∗^*p* < 0.05, one-way ANOVA followed by Bonferroni's post hoc test analysis for multiple comparison. Scale bar = 100 *μ*m.

The evaluation of cell migration showed that growth d-ECM significantly promoted ASC migration compared with adipogenic d-ECM or TCP (Figures 4(a) and 4(b)). However, cells reseeded on adipogenic d-ECM migrated less than cells grown on TCP or on growth d-ECM. To test the ability of d-ECM to promote differentiation of ASCs, lipid droplets were stained with Oil Red O. Small droplets were visible in ASCs cultured on adipogenic d-ECM. The droplets were visually different from those observed in ASCs differentiated using adipogenic culture medium. In contrast, no positive staining was detected in cells cultured on growth d-ECM or TCP (Figure 4(c)). We further tested the mRNA expression levels of the adipogenic genes fatty acid binding protein 4 (FABP4) and peroxisome proliferator-activated receptor *γ* (PPAR*γ*). After culturing for 7 days, the expression levels of FABP4 and PPAR*γ* were significantly higher in cells grown on adipogenic d-ECM than those grown on growth d-ECM or TCP (Figures 4(d) and 4(e)).

### 4.5. Integrin Expression in ASCs Cultured on Decellularized ECM

**Figure 5:**
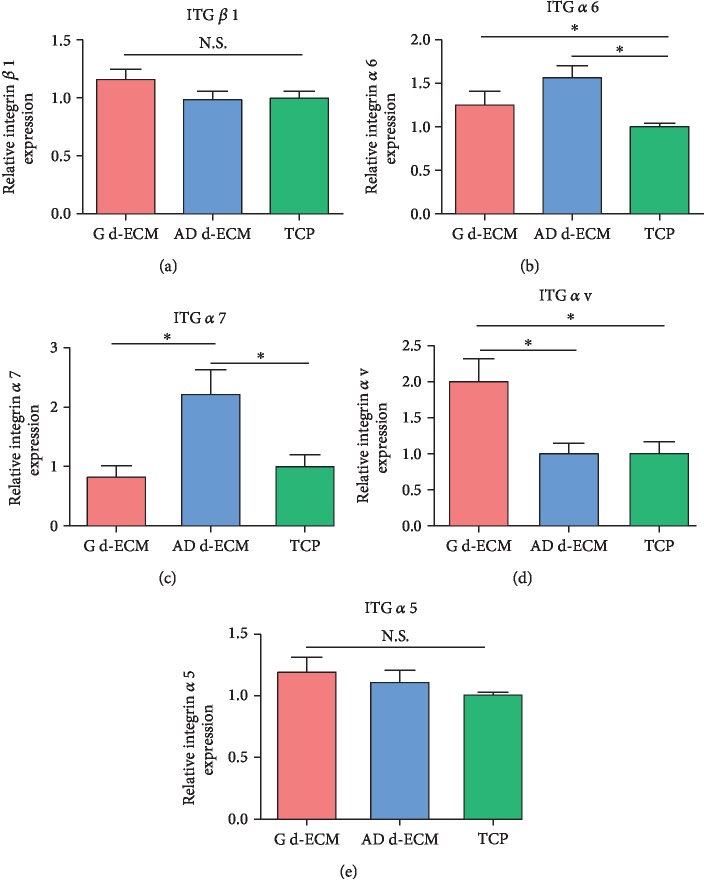
Gene expression of integrins (*α*5, *α*V, *α*6, *α*7, and *β*1) produced by ASCs reseeded on three different substrates. qRT-PCR analysis of (a) integrin *α*5, (b) integrin *α*V, (c) integrin *α*6, (d) integrin *α*7, and (e) integrin *β*1 mRNA expression in growth d-ECM, adipogenic d-ECM, and TCP. Results are presented as the mean ± SD. ^∗^*p* < 0.05, one-way ANOVA followed by Bonferroni's post hoc test analysis for multiple comparison.

Interactions between cells and the ECM were evaluated using qRT-PCR, quantifying the expression of integrin protein genes in ASCs reseeded on d-ECM and TCP during the 7-day culture (Figure 5). Higher production of surface integrin *α*6 was observed when cells were reseeded on growth d-ECM and adipogenic d-ECM compared to TCP. The expression of integrin *α*7 was increased in ASCs cultured on adipogenic d-ECM, but not in those ASCs cultured on TCP or growth d-ECM. Furthermore, the expression of integrin *α*V was significantly increased in ASCs cultured on growth d-ECM compared to that in cells cultured on TCP or adipogenic d-ECM. The expression of integrins *α*5 and *β*1 was not significantly different among the three groups.

## 5. Discussion

Stem cells can secrete ECM proteins and components, resulting in ECM remodeling and optimization to produce critical biochemical and physical signals [16]. The ECM microenvironment can also control the fates of stem cells [6, 17]. The ECM contains a variety of protein components which can regulate cell phenotype via assembly of integrins, focal adhesions, and cytoskeletal reorganization, ultimately regulating cell behaviors such as migration, proliferation, and differentiation [18, 19]. In this study, we found that ECM secreted by ASCs changed dynamically from fibronectin-rich to laminin-rich during differentiation of ASCs. These changes in ECM composition resulted in changes in the cellular behaviors of ASCs reseeded on these matrices.

Previous studies have shown that the extracellular microenvironment can dynamically change to regulate stem cell differentiation [20]. ASCs can undergo dynamic adipogenic lineage differentiation. To determine whether ECM components secreted by ASCs changed during the adipogenic differentiation, ECM composition was evaluated using immunofluorescence analysis. Our results showed that fibronectin was abundant in the ECM produced by undifferentiated ASCs but was only detected at low levels at the late stages of ASC differentiation. In contrast, laminin gradually increased during the 14-day adipogenic differentiation period. These results indicated that the ECM protein composition remodeled during *in vitro* adipogenesis. Adipogenesis is a two-phase process in which the ECM transitions from a fibrillar to a laminar structure as cells move from the growth phase to the differentiation phase, which is characterized by storage of large amounts of triglycerides [21].

In this study, we found that total protein content was significantly decreased following decellularization of the ECM [13, 22]. However, decellularization did not remove ECM proteins. These results suggested that the proteins removed were primarily intracellular proteins. We also showed that decellularization almost completely removed cytokines and showed that ASCs reseeded on d-ECM were not affected by residual cytokines.

The extracellular matrix (ECM) plays a key role in cell regulation during the adipogenesis. Cytokines can bind with ECM that provide structural and biochemical support to the surrounding cells. These multi-microenvironments are important in regulating cell migration, proliferation, and differentiation. In our study, the amounts of growth factors tested were decreased during the decellularization process. Therefore, we have basically ruled out the effect of cytokines on cell behavior. We hypothesized that the protein components of the extracellular matrix regulate cell behaviors. After all, the protein composition of ECM, especially fibronectin and laminin, changes significantly during ASC lipogenesis. To assess the effects of ECM remodeling on cell behavior, we reseeded ASCs onto the growth d-ECM and the adipogenic d-ECM. The results showed that ASCs reseeded on the adipogenic d-ECM exhibit more flat and rounded morphology than cells reseeded on growth d-ECM. Furthermore, the ASCs reseeded onto d-ECM exhibited more spreading and cellular extension than cells plated on TCP. These results indicated that d-ECM can regulate the cytoskeletal structure of ASCs. High cytoskeletal polarity promotes cell proliferation and migration, while low polarity indicates that cells are static and more likely to undergo adipogenic differentiation [23]. Therefore, we examined the effects of the different ECM substrates on the proliferation of ASCs. Our findings showed that the proliferation rate was not significantly different among the three groups. However, previous studies showed that ECM secreted from cells facilitated the expansion of MSCs [24]. However, ECM secreted by ASCs did not enhance proliferation in our study. Interestingly, colony-forming assay showed that ASCs grown on d-ECM formed more colonies, particularly on growth d-ECM. We also evaluated cell migration of ASCs reseeded onto d-ECM and showed that growth d-ECM significantly promoted cell migration. Previous studies showed that fibronectin can promote cell migration through integrin *α*v-activated signaling and actin filament formation [25]. Our results suggested that fibronectin-rich growth d-ECM promoted cell migration.

To assess the ability of d-ECM to trigger differentiation of ASCs, we cultured reseeded ASCs for 7 days. Oil Red O staining showed that lipid accumulation occurred to a greater extent in ASCs cultured on laminin-rich adipogenic d-ECM. No positive Oil Red O staining was observed in ASCs cultured on growth d-ECM or TCP. To further evaluate the effects of d-ECM on adipogenic differentiation of ASCs, the gene expression levels of the adipogenic markers FABP4 and PPAR*γ* were measured. The expression levels of FABP4 and PPAR*γ* in ASCs grown on adipogenic d-ECM were higher than those in ASCs grown on growth d-ECM or TCP. As adipogenic d-ECM was laminin-rich and laminin has been shown to enhance adipogenesis [12, 26], our results suggested that laminin induced the expression of PPAR*γ* and FABP4.

Interactions between ASCs and ECM integrins have been widely reported [18, 19]. Similar to receptors for paracrine signals, integrins transduce signals from the extracellular matrix to regulate gene expression and cellular function [27, 28]. In our study, integrins *α*5 and *α*V (binding with fibronectin) and *α*6 and *α*7 (binding with laminin) were investigated. These integrins were previously shown to play an important role in the control of cell behaviors [24, 25]. Our results showed that integrins *α*6 and *α*7 were upregulated in ASCs reseeded on adipogenic d-ECM, suggesting that laminin in d-ECM may have interacted with integrin receptors during ASC adipogenesis. Interestingly, the expression of integrin *α*7 was higher in ASCs reseeded on adipogenic d-ECM than that on growth d-ECM. However, no studies have reported that integrin *α*7 regulates adipogenesis of ASCs. Further studies are required to evaluate the effects of laminin binding to integrin *α*7 on adipogenic differentiation of ASCs. Taken together, these results suggested that the adipogenic d-ECM did play a role in triggering the presentation of integrins *α*6 and *α*7, which in turn affected subsequent cell material interactions.

In addition, the expression of integrin *α*v was significantly higher in cells reseeded on growth d-ECM than in those seeded on TCP or adipogenic d-ECM, suggesting that integrin *α*V interactions with fibronectin may facilitate migration of ASCs. Previous studies showed that integrins *α*5 and *α*v play important roles in cell proliferation [29, 30]. However, our results did not show any differences in proliferation between ASCs reseeded on growth d-ECM, which had significantly higher expression of integrin *α*V, and ASCs grown on adipogenic d-ECM. These results suggested that integrin *α*v may play an important role in cell migration.

## 6. Conclusions

In conclusion, we demonstrated that ECM secreted during stepwise adipogenesis played an important role in migration and differentiation of ASCs. During adipogenic differentiation, ECM secreted from the cells changed dramatically from fibronectin-rich to laminin-rich. In addition, ASCs showed diverse cellular functions when reseeded on different decellularized ECM. ASCs reseeded on fibronectin-rich growth d-ECM showed increased migration. In contrast, adipogenesis of ASCs was enhanced by laminin-rich adipogenic d-ECM. Moreover, integrins *α*6, *α*7, and *α*V were produced by ASCs reseeded on growth and adipogenic d-ECM, which indicated that these integrins play an instrumental role in cellular adipogenesis and migration. This study demonstrated the important role of ECM secreted by cells on migration and differentiation of ASCs and provides a strategy to achieve precise regulation of stem cell function in adipose tissue engineering.

## Figures and Tables

**Table 1:** List of target genes and their primer sequence used in this study.

Primers	Sequence
PPAR*γ*	F: 5′-CAATCTGCCTGAGGTCTG-3′
	R: 5′-TGGAGCCTAAGTTTGAGTT-3′
FABP4	F: 5′-GACTTTCCATCCCACTTC-3′
	R: 5′-AAACACCGAGATTTCCTT-3′
ITG*α*5	F: 5′-GAGGATTCCAGTCGCTGA-3′
	R: 5′-GAAGGCAGGCACCAGTAT-3′
ITG*α*6	F: 5′-AGCCTTGTGGTAGGTGGC-3′
	R: 5′-CAGGGATAGCGTGGTGGA-3′
ITG*α*7	F: 5′-AGAGGCATTCTCGTTGGA-3′
	R: 5′-ATGGGACCGCCCTGTTTG-3′
ITG*α*V	F: 5′-CACTGGAGGTTCAGGATT-3′
	R: 5′-GCTGCCGTTGAGATAAGA-3′
ITG*β*1	F: 5′-GGTGAAATAGAACCAGCAGT-3′
	R: 5′-AAAGTGGAAAGCAGGGAG-3′
GAPDH	F: 5′-TCTCTGCTCCTCCCTGTTC-3′
	R: 5′-ACACCGACCTTCACCATCT-3′

## Data Availability

The data used to support the findings of this study are available from the corresponding authors upon reasonable request.

## Supplementary Materials

Supplementary Materials Figure S1: differentiation of ASCs. (a) Rat adipose tissue-derived stromal cells (ASCs) were maintained and expanded in growth medium. Bar: 100 *μ*m. (b) ASCs were cultured in adipogenic medium and differentiated into adipocytes over 14 days. Scale bar: 100 *μ*m.
