# Supplementary material for: Stepwise Adipogenesis of Decellularized Cellular Extracellular Matrix Regulates Adipose Tissue-Derived Stem Cell Migration and Differentiation
Source: Stem Cells Int. 2019 Nov 6;2019:1845926. doi: 10.1155/2019/1845926 (PMC6875313; doi:10.1155/2019/1845926)
Supplement: Supplementary Materials — Figure S1: differentiation of ASCs. (a) Rat adipose tissue-derived stromal cells (ASCs) were maintained and expanded in growth medium. Bar: 100 μm. (b) ASCs were cultured in adipogenic medium and differentiated into adipocytes over 14 days. Scale bar: 100 μm. [file 1845926.f1.pdf]

Supplemental information

Stepwise adipogenesis of decellularized cellular extracellular matrix regulates adipose tissue-derived stem cell migration and differentiation

Ziang Zhang[1,2], Rongmei Qu[1], Tingyu Fan[1], Jun Ouyang[1], Feng Lu[2☆], Jingxing Dai[1☆]

Figure. S1. Differentiation of ASCs. (a) Rat adipose tissue-derived stromal cells (ASCs) were maintained and expanded in growth medium. Bar, 100 μm. (b) ASCs were cultured in adipogenic medium to and differentiated into adipocytes over 14 days. Scale bar = 100 μm.

Growth Adipogenic

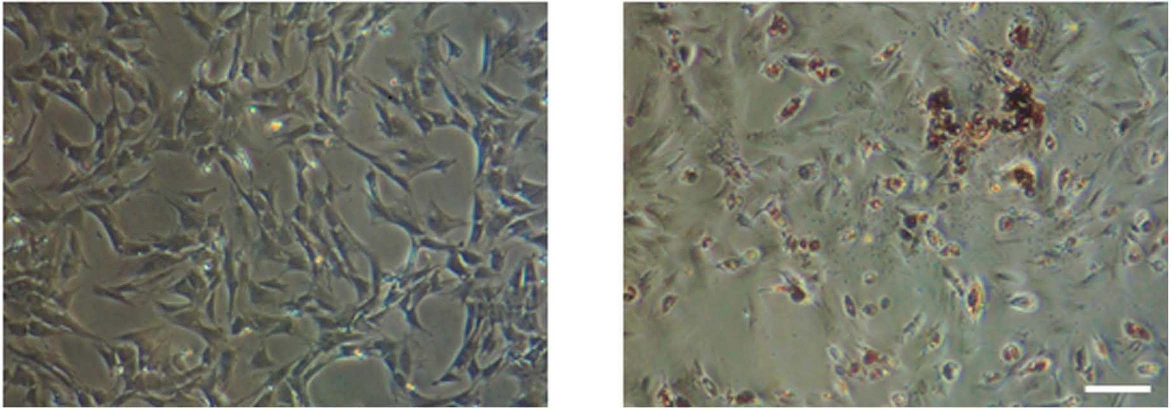

(a) (b)
